# Molecular Interactions of Arterial Hypertension in Its Target Organs

**DOI:** 10.3390/ijms22189669

**Published:** 2021-09-07

**Authors:** Joanna Kućmierz, Weronika Frąk, Ewelina Młynarska, Beata Franczyk, Jacek Rysz

**Affiliations:** Department of Nephrology, Hypertension and Family Medicine, Medical University of Lodz, ul. Żeromskiego 113, 90-549 Łódź, Poland; joanna.kucmierz@interia.pl (J.K.); frweronika@gmail.com (W.F.); bfranczyk-skora@wp.pl (B.F.); jacek.rysz@umed.lodz.pl (J.R.)

**Keywords:** arterial hypertension, blood pressure, renin-angiotensin-system, vascular remodeling, reactive oxygen species, matrix metalloproteinases

## Abstract

Arterial hypertension (AH) is a major risk factor for the development of cardiovascular diseases. It is estimated that the disease affects between 10% and 20% of the adult population and is responsible for 5.8% of all deaths worldwide. Several pathophysiologic factors are crucial in AH, including inappropriate activation of the renin-angiotensin-aldosterone system, oxidative stress and inflammation. The heart, kidney, brain, retina and arterial blood vessels are prime targets of hypertensive damage. Uncontrolled and untreated AH accelerates the damage to these organs and could cause their failure. Damage to these organs could also manifest as coronary heart disease, cognitive impairment, retinopathy or optic neuropathy. For better understanding, it is important to analyze molecular factors which take part in pathogenesis of AH and hypertension-related target organ damage. In our paper, we would like to focus on molecular interactions of AH in the heart, blood vessels, brain and kidneys. We focus on matrix metalloproteinases, the role of immune system, the renin-angiotensin-aldosterone system and oxidative stress in hypertensive induced organ damage.

## 1. Introduction

Arterial hypertension (AH) is a chronic medical condition in which blood pressure is elevated and poses a major risk factor for the development of cardiovascular diseases [1]. It is estimated that the disease affects between 10% and 20% of the adult population and is responsible for 5.8% of all deaths worldwide [2].

Arterial hypertension could be diagnosed when the blood pressure (BP) value is 140/90 mmHg or higher. BP is very variable, so the diagnosis of arterial hypertension should not be determined on the basis of a one-time set of BP measurements over a period of one medical visit, except where BP values are very elevated (stage 3 of AH). Arterial hypertension should be confirmed by at least two readings on two or more separate occasions [3,4,5]. Depending on the BP values, AH could be divided into three categories (Table 1).

In more than 90% of cases, AH has an unknown etiology and is diagnosed as a primary AH; however, the pathogenesis of AH is better understood nowadays thanks to available data on a certain number of risk factors. The remaining 10% of cases are diagnosed as secondary AH and characterized by autonomous aldosterone production, caused by renal, cardiovascular, neurological and endocrine diseases (Table 2) [6].

There are many potentially modifiable risk factors for AH, for instance, overweight/obesity, physical inactivity, poor diet, low folate intake [8], excessive sodium intake, excessive alcohol intake and alcohol abstinence; however, the relationship between hypertension and alcohol remains unclear [2]. In addition, cigarette smoking induces a hypertensive effect, mainly through the stimulation of the sympathetic nervous system [9]. It is worth emphasizing that there are genetic determinants in the direction of arterial hypertension and interaction with environmental factors determines how severe the rise of blood pressure will be [10].

It is not only risk factors that are important in the development of AH. Several pathophysiologic factors are crucial in AH, including inappropriate activation of the renin-angiotensin-aldosterone system (RAAS), oxidative stress and low-grade inflammation, which leads to production vasoactive molecules and causes other changes in function and structure of blood vessels which are described below. Other factors include impaired insulin-mediated vasodilatation in hyperinsulinism, excessive activation of the sympathetic nervous system and abnormal sodium handling by the kidney [11].

Treatment of AH is both pharmacological and non-pharmacological and those two types of therapy should be led simultaneously to decrease cardiovascular risk. Lifestyle interventions can undoubtedly lower BP; however, most patients with hypertension will also require drug treatment [3]. According to the guidelines, blood pressure should be normalized within 3 months of its diagnosis. It is recommended that the goal of therapy among all patients should be a reduction in BP to <140/90 mm Hg. If the treatment is well tolerated, it should be targeted to 130/80 mmHg or lower in most patients [3] In patients <65 years it is recommended that SBP should be lowered to a BP range of 120–129 mmHg in most patients and after 65 years-to a 130–139 mmHg.

Pharmacological treatment is based on known pathophysiological processes like excessive activation of renin-angiotensin-aldosterone system. Guidelines recommend angiotensin-converting-enzyme inhibitors (ACE-I), angiotensin receptor blockers (ARBs), beta-blockers, thiazides and thiazide-like diuretics and calcium channel blockers (CCBs) as drugs of first choice in different combinations [6].

ACE-I and ARBs are the most widely used medicines in the treatment of AH and are characterized by similar effectiveness; however, usage of ARBs is associated with a significantly lower number of patients discontinuing treatment due to side effects compared to the other classes of drugs. Both groups reduce the risk of diabetic nephropathy; however, the use of ACE-I and ARBs at the same time increases the risk of kidney damage [4].

Beta-blockers decrease the frequency of heart failure, strokes and other serious cardiovascular events. They are advised for patients suffering from ischemic heart disease, heart failure, arrhythmias and after myocardial infarction. It should be noted that it is forbidden to combine beta-blockers with verapamil/diltiazem due to the serious risk of bradycardia [4].

Thiazides and thiazide-like diuretics are especially effective among patients with AH and heart failure, but could decrease potassium level in the serum and cause insulin resistance. Due to the mechanism of action, they are contraindicated among patients with eGFR < 45 mL/min [4].

Calcium channels blockers strongly reduce risk of stroke but are less effective in prevention of heart failure than other groups of drugs.

Non-pharmacological treatment involves a low-salt diet-under 5 g per day, adequate potassium intake, avoidance of excessive alcohol consumption (binge drinking) and implementation of days without any alcohol, smoking cessation, physical exercise (at least 30 min moderate dynamic physical effort 5–7 days a week) and weight loss [6]. People with hypertension should have a healthy balanced diet containing vegetables, legumes, fresh fruit, low-fat dairy products, whole grains, fish and unsaturated fatty foods (especially olive oil), as well as reducing the consumption of red meat and saturated fatty acids [4].

The heart, kidney, brain, retina and arterial blood vessels are prime targets of hypertensive damage. Uncontrolled and untreated AH accelerates the damage to these organs and could cause their failure [12,13]. Hypertension is the second most important cause of CKD after diabetes and increases the prevalence of brain damage such as transient ischemic attack (TIA) and stroke which are the most dramatic acute clinical manifestations of AH [3]. Damage to these organs could also manifest as coronary heart disease, cognitive impairment, retinopathy or optic neuropathy (Table 3) [13].

Due to many complications of AH, every patient with AH should be regularly diagnosed in the direction of above diseases. Additional tests should include 12 lead ECG, levels of albumin, urea and creatinine in the serum, fundus examination in patients with grade 2 or 3 hypertension or hypertensive patients with diabetes, estimated glomerular filtration rate, echocardiography, abdominal USG in the direction of renal diseases and assessment of cognitive functions [14]. Table 3: Hypertension-related target organ damage [13].

In our paper, we focus on the molecular interactions of AH in the heart, blood vessels, brain and kidneys.

We focus on matrix metalloproteinases (MMPs) as a group of enzymes which could change the function of endothelium vascular smooth muscle and cardiomyocytes. Those changes could provide to ischemic stroke, myocardial infraction and other cardiovascular and systemic diseases [15,16,17]. Furthermore, during the past few years, it has become apparent that cells of the immune system also contribute to this disease. We would like to demonstrate that immune cell infiltration of the vessel wall, kidney and central nervous system participates in the development of hypertension and in hypertensive organ injury.

The renin-angiotensin-aldosterone system (RAAS) has key regulatory functions for blood pressure and fluid homeostasis. The overactivation of the RAAS axis promotes inflammation, via activation of the immune system. Moreover, an increased level of Ang II leads to vascular and renal injury, sodium retention, endothelial dysfunction. Therefore, the RAAS act on rising arterial blood pressure. Our review will focus on their effects on hypertensive organ damage.

The other important mechanism is organ damage via oxidative stress which is characterized by modifications in the oxidant/antioxidant ratio. Those modifications lead to a disruption of oxidation-reduction (redox) signaling. Oxidative stress is a process induced mostly by an excessive amount of reactive oxygen species (ROS) and is strongly relevant to low-grade or persistent inflammation and angiotensin II activation.

## 2. Matrix Metalloproteinases and Hypertension-Mediated Organ Damage

Matrix metalloproteinases (MMPs) are important extracellular enzymes which belong to the zinc-dependent endopeptidases and could be secreted by fibroblasts, vascular smooth muscle and leukocytes [15,16]. The MMP family is divided into six categories: collagenases, gelatinases, stromelysins, matrilysins, membrane-type MMPs and others which are not classified MMPs [17].

Increased interest in inflammation has investigated new inflammatory mediators because MMPs expression/activity could be influenced not only by hormones or growth factors. The activity of MMPs increases also during the inflammatory process because MMPs are secreted by pro-inflammatory cells and their secretion is stimulated by cytokines [15].

They degrade various proteins in the extracellular matrix (including collagen and elastin) and are involved in many physiological and pathological processes [15,16]. Many researchers observed a noticeably higher level of MMP-9 in the serum of hypertensive patients compared to normotensive controls [15].

They also have influence on function of endothelium, vascular smooth muscle cells and could stimulate changes in cardiomyocytes [15].

MMPs activity could cause vascular remodeling and changes in ECM and VSM which may be associated with AH. Some researchers argue that vascular remodeling could be the first step in the development of cardiovascular complications including atherosclerosis, stroke, heart and renal failure [15]. It is worth emphasizing that MMPs are key factors in the response of the immune system and vascular inflammation by activations of MMP-1 which degrades collagen and gelatin [15].

In pathogenesis of AH, one of the most crucial factors are changes in function of VSM which include cell migration, Ca^2+^ signaling, contraction and proliferation of VSM cells. Proliferation is regulated by MMPs in several mechanisms including proteolytic lysis of growth factors so that they become available to cells of VSM. MMPs also regulate degradation of ECM and functioning of the receptors which oversee cell migration [18]. 

Moreover, MMPs mediate the effects of several pro-angiogenic factors like FGF, TGF-α, TGF-β, VEGF (vascular endothelial growth factor) and angiogenin so that they are important regulators of angiogenesis [15].

It is worth emphasizing that MMPs have recently been associated with receptor cleavage of the receptor for the insulin. It could lead to insulin resistance which is typically associated with hypertension [19].

From taking part in those processes and research on animal and people studies, we ascertain the important influence of MMPs in the pathogenesis of HA and organs damage [15].

Some of the studies revealed an association between MMPs and acute coronary syndrome manifested by elevated level of MMP-2 and MMP-9 versus in stable coronary artery disease [15,20].

Polymorphisms in the MMP-1, MMP-2, MMP-3, MMP-9 and MMP-12 genes are associated and could have impact on development of ischemic stroke by damage of the blood–brain barrier and reduction of levels of the endogenous proteases tissue-type plasminogen activator.

Moreover, elastolytic function of MMP-2 could lead to subsequent weakening of the aortic wall and-in the result-cause thoracic aortic aneurysm (TAA) or abdominal aortic aneurysm (AAA) [15].

**Figure 1:**
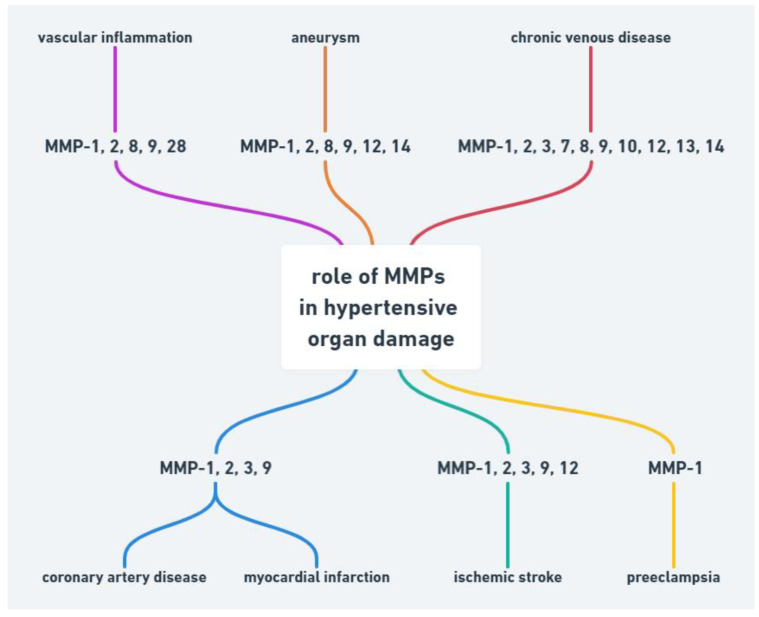
Role of MMPs in hypertensive organ damage.

Abnormality in MMPs function may be also associated with other cardiovascular diseases such as hypertension, atherosclerosis, aneurysm, chronic venous disease and preeclampsia (Figure 1).

The relative risk of renal damage in patients with AH is lower than other cardiovascular complications; however, researchers demonstrated that MMP-2 and MMP-9 could be involved in abnormal tissue remodeling associated with AH in renal disease by pathogenic remodeling of ECM, leading to the nephrosclerosis and, finally, to the chronic kidney disease (CKD) [21].

## 3. Role of Immune System in Hypertension and Target Organ Damage

**Figure 2:**
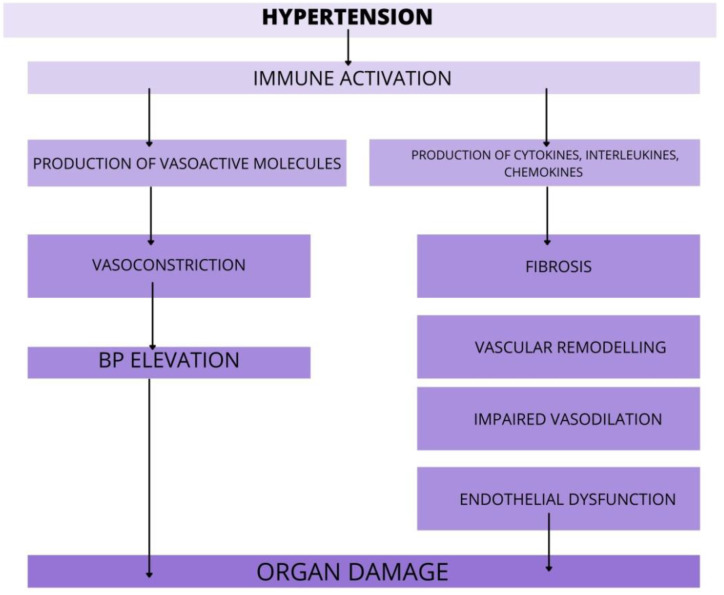
The role of immune cells in target organ damage. In response to hypertensive stimuli, the immune system is activated. Various immune cell subsets release pro-hypertensive molecules that promote organ damage via action in vasculature, kidneys, heart, and brain. Furthermore, the immune cells rise the BP via enhanced vasoconstriction and by that, worsen the course of AH. Abbreviations: BP-blood pressure, AH—arterial hypertension.

In the past few years, evidence has accumulated to suggest that hypertension is, at least in part, an immune-mediated inflammatory disorder. AH is associated with the accumulation of immune cells into kidneys, vasculature, heart or brain, where they mediate tissue injury, impede vascular relaxation and enhance sodium reabsorption. There is evidence from several studies, that various immune cell subsets infiltrating organs during hypertension and augment the progression of organ damage (Figure 2).

It is now widely acknowledged that low-grade or persistent inflammation is a key player in the development and maintenance of AH. The compounds of the immune system are managed to produce vasoactive molecules, such as Ang II, endothelin-1 or prostaglandins. All of them are known as the hypertensive mediators and TOD mediators [22]. These data showed that monocytes and macrophages enhance vasoconstriction and sodium retention. Furthermore, antigen-presenting cells -the dendritic cells (DCs) and B lymphocytes may stimulate the rise of BP by modulating the T cell function. Furthermore, the DCs produce mediators like IL-1β and IL-6, which could modify BP independently of T cells [23,24]. On the contrary, Il-10 has a protective role on renal damage by reducing albuminuria. Furthermore, Il-10 inhibits endothelial dysfunction and superoxide production. These findings raise the possibility that disrupting inflammatory functions could restrain target organ damage, accruing from hypertension through blood pressure-independent mechanisms. This study aimed that after administration of Ang II, the mice with MR deficiency in T-cell were associated with lower BP, both systolic and diastolic. Moreover, there was decreased renal and vascular dysfunction. Therefore, the mineralocorticoid receptor (MR) antagonists are used in AH treatment for many years, the blockage of MR in T-cells could be a novel target in antihypertensive therapies [25]. In the vasculature, the immune cells are accumulating in the perivascular fat and by producing the various molecules (e.g., cytokines, chemokines, MMPs, ROS); they leading to the BP elevation, impairing vasodilation, stimulating collagen synthesis and degrading the elastic lamina, as well as increasing vascular stiffness [26]. Moreover, there are studies highlighting that, due to hypertension, there is the accumulation of activated Th17 cells in the cardiac wall. The Th17 cells in the heart lead to remodeling and fibrosis and therefore, to cardiac hypertrophy, arrhythmia or heart failure. The Th17 cells also produce Il-17-, the proinflammatory cytokine, especially in high salt conditions [27]. The Il-17 increases the BP pressure through inhibiting endothelial nitric oxide production, increasing ROS formation and promoting fibrosis. Interestingly, if the level of Il-17 is lowered, it aids in the BP’s normalization and reduces the inflammatory response in the myocardial tissue [28]. Furthermore, Il-17 has a major role in aortic stiffening, because of the increased synthesis and deposition of collagen within the aortic wall [29]. Moreover, the Il-17 is also the mediator of renal injury. This cytokine is enhancing renal sodium retention and glomerular injury. The therapies targeting Il-17 might be an effective treatment for hypertension and its associated TOD [27]. Furthermore, these results discover that the Il-11 expression in adventitial fibroblasts is stimulating the adventitial remodeling and vascular fibrosis. The knockout of Krüppel-like factor-15 expression, which promotes the Il-11 production, might be another target for decreasing the vascular remodeling in AH [30]. It is important to note that the accumulation of immune cells within the tissue leads to local production of hypertensive mediators, such as Ang II, and increase the local levels of hypertensive stimuli within the kidneys, vasculature or nervous system [22]. Lately, extensive evidence demonstrates the role of brain perivascular macrophages (PVMs) in the development of AH. The pro-inflammatory mediators, such as IL-1β, increase PGE2 expression within PVMs, which results in enhanced sympathetic activation and BP elevation [31]. Furthermore, the pericytes and the astrocytes in the blood–brain barrier (BBB) could also be affected by systemic inflammation. As a consequence, the impairment of cells in the BBB leads to increased level of immune cells within the brain and, therefore, to the neuronal overexpression of PVMs [32]. Cytokines that are particularly relevant to hypertension are produced by T cells, B cells, mast cells, macrophages and DCs. In these series of studies, the authors discussed the role of chemokines in the pathophysiology of hypertension. They indicate that tissue expression of CCL2 and CCL5 is raised in hypertension. Circulating levels of CCL2 are upregulated in hypertensive patients and correspond with the degree of hypertension-associated organ damage. Moreover, the CCL2/CCR2 axis consistently exacerbates hypertensive tissue injury and inflammation. Although CCL5 expression increases in the kidney during hypertension, its effects on renal inflammation and injury seem to be protective [33]. There are studies that indicate the role of the CXC cytokines family in hypertension. These molecules decrease vascular dysfunction and remodeling, as well as reducing the progress of fibrosis. However, these data require further study.

These studies discovered the roles and mechanisms of NLRP3 inflammasome in hypertension. NLRP3 is a protein complex and a vital component of the immune system, and its activation is a major mediator of inflammatory response via caspase-1 activation. In hypertension, various mediators lead to the NLRP3 inflammasome formation and activation. The NLRP3 is a major modulator of inflammation and organ damage, by activation of caspase-1 and promoting a type of cell death called “pyroptosis’’ [34,35]. NLRP3 knockdown reduces blood pressure, inhibits vascular smooth muscle cells inflammation and attenuates vascular remodeling. NLRP3 may be a novel target for the intervention of hypertension and vascular remodeling [36,37].

Additionally, investigations on the immune reactivity in hypertension might result in the identification of new strategies for the treatment of the disease.

## 4. Role of Renin-Angiotensin-Aldosterone-System (RAAS) in Hypertension and Target Organ Damage

The dysregulation of renin-angiotensin-aldosterone-system (RAAS) plays a critical role in the pathophysiology of hypertension and in the occurrence of the TOD. Decreased levels of intratubular sodium, hypotension in the afferent arterioles of the renal glomerulus and sympathetic activation affect the synthesis and release of inactive renin by the kidney. In the bloodstream, the renin is activated and as its consequence, it leads to the breakout of angiotensinogen to Ang I. Finally, due to the angiotensin-converting enzyme, Ang I is converted to Ang II-the major component of RAAS.

To begin with, in these data, it was demonstrated that RAAS can be successfully suppressed by renin inhibitors. This is why it could be a new potential therapy for hypertensive treatment and, furthermore, to reduce TOD. It has been shown that renin inhibitors, in particular, help prevent renal or eye damage [38]. In the brain, renin is synthesized in astrocytes. Hyperactivation of renin signaling disable cognitive function due to overactivation of RAAS axis [39]. Renin inhibition could be combined with other drugs, such as ACE inhibitors or angiotensin–1 receptor (AT-1R) blockers, to block both Ang II and aldosterone generation. Clinical studies evaluating the benefits of renin inhibition are ongoing and the results are encouraging [38].

**Figure 3:**
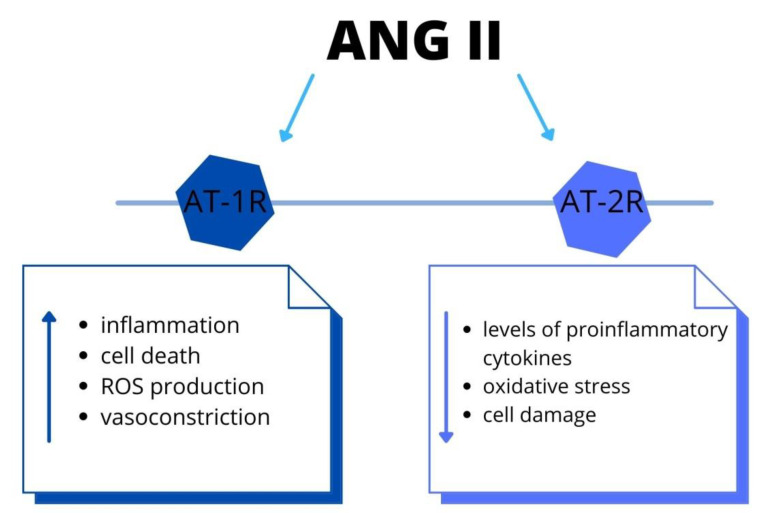
The RAAS system: the biological effects via angiotensin II-type 1 and type 2 receptors activation. Both systemically and locally produced Ang II bind to AT-1R and promote inflammation and cell death. Recent studies have shown that Ang II-induced activation of AT-2R and elicits the opposite functions to those of AT-1R. Abbreviations: Ang II-angiotensin II, AT-1R-Ang II type 1 receptor, AT-2R-Ang II type 2 receptor, ROS-reactive oxygen species.

Ang II (angiotensin II), a main pro-hypertensive hormone, mediates target organ disorder, by activation of immune cells [40]. Moreover, the leukocyte ligand-P-selectin glycoprotein ligand-1 (Psgl-1) manages endothelial function and mediates the IL-17 production in order to Ang II. Due to Psgl-1, there are increased levels of inflammatory biomarkers. The accumulation of immune cells leads to endothelial dysfunction and, thus, blood pressure elevation. Therapeutic targeting of Psgl-1 or downstream IL-17 could be a target in certain anti-hypertensive therapy [41]. Furthermore, Ang II causes endothelial dysfunction due to vasomotor alteration and migration and attachment of leukocytes [42]. These data showed the link between alteration in the angiotensinogen gene and hypertension. The authors demonstrated that Ang II acts not only via the AT-1 receptor but there is also the intrarenal synthesis of Ang II. Moreover, intrarenal Ang II stimulates the angiotensinogen mRNA levels. Thus, there is a positive feedback mechanism, by which the production of angiotensinogen mRNA increases further the production of intrarenal Ang II. The elevation of intrarenal Ang II results in renal and vascular injury, triggering the progression of AH [43]. It is essential to indicate that also the central nervous system (CNS) has a part in AH pathogenesis. There is a secretion of Ang II in neuronal tissue, which acts locally on raising the arterial blood pressure, as well as activating the immune cell within the CNS. However, the complete understanding of these mechanisms is still unknown and further research to acknowledge this is required [44]. In addition, the RAAS has a major role in the pathophysiology of traumatic brain damage (TBD), via AT-1R and AT-2R signaling. The AT-1R mediates inflammation, cell death, oxidative stress and vasoconstriction in the brain. On the contrary, the AT-2R decreases levels of proinflammatory cytokines, oxidative stress and improves cell survival. This could be a novel target for treating TBD [45]. Furthermore, Ang II stimulate production of proinflammatory molecules and increase ROS via AT-1R pathway [45] (Figure 3). Therefore, this signaling is associated with aggravated risk of several diseases, including stroke, retinopathy and coronary artery disease. Within the brain, the Ang II regulates the cerebral blood flow and neuroinflammation [46]. In the cardiovascular system, the Ang II leads to vasoconstriction, sodium–water retention, inflammation, hypertrophy and fibrosis. Therefore, it is associated with the development or progression of cardiovascular and renal disease [47]. In addition, according to these data, the reduced modulation of RAAS activity in response to sodium restriction and increased aldosterone on a high sodium diet might predisposed hypertensive patients to abnormal cardiac remodeling [48].

On the contrary to the damaging actions of Ang II, there are developing data that indicate that there is another product of the RAAS: the angiotensin (1–7) (Ang (1–7)). The Ang (1–7) mediates the cardiovascular protective effects. Furthermore, this peptide attenuates the cerebral damage and has a cerebroprotective effect during ischaemic stroke [49]

Aldosterone, a component of the RAAS, maintains the balance of liquid volume and electrolyte levels in the human body; however, its overexpression may induce myocardial and vascular fibrosis and chronic kidney disease progression [50]. This study indicated that vascular remodeling (VR) due to hypertension leads to alteration in vascular structure and function. This study showed the protective role of microRNAs (MiR-26a) in VR, which could be investigated as a promising target in the therapy of hypertensive VR [51].

The kallikrein–kinin system (KKS) works as a regulatory system against the RAAS. It has been demonstrated that KKS does not play a significant role in hypertension and its pathophysiology; however, Hamid et al. (2020) indicated that KKS increases the anti-hypertensive effect of ACE-I. Furthermore, it has a beneficial effect on the kidneys, heart and vascular system and, through this, decreases the progression of TOD [52].

The sodium/hydrogen exchanger 3 (NHE-3) manages the entry of Na+ into and the exit of H+ from the proximal tubules and, through this, it is responsible for reabsorbing about 50% of filtered Na+ and about 80% of filtered HCO3- in the proximal tubules of the kidney. The authors of this study hypothesized that overexpression of NHE-3 might have a role in salt and fluid retention; therefore, it could impact BP. Interestingly, they found that upregulation of these transporters in mice might be a potential way of developing AH, as the fluid and salt balance is disturbed [53].

These studies discovered one of the mechanisms of TOD due to hypertension and indicated the RAAS as its major cause. These findings suggest a potential new tool for better treatment that might reduce TOD.

## 5. Role of Oxidative Stress in Hypertension and Hypertensive Organ Damage

Oxidative stress is a process induced mostly by an excessive amount of reactive oxygen species (ROS) (Table 4) and is characterized by modifications in the oxidant/antioxidant ratio, which leads to a disruption of oxidation reduction (redox) signaling and molecular damage [54,55].

ROS are reactive molecules that play key roles in the regulation of many cell functions and in both physiology and pathophysiology processes [54,56]. The sources of cardiovascular ROS include respiratory burst of phagocytes (granulocytes, monocytes, macrophages), production in peroxisome, nonphagocytic NADPH oxidases (Nox), mitochondrial oxidases, xanthine oxidase, endoplasmic reticular oxidases and uncoupled nitric oxide synthase (NOS) [55,57]. Mitochondrial dysfunction and generation of the ROS in the brainstem are crucial in neurogenic hypertension [58].

**Table 4:** Examples of ROS with the most important influence in the cardiovascular system [54,56,59].

Name of ROS	Chemical Formula
Superoxide	O_2_^▪−^
Hydrogen peroxide	H_2_O_2_
Hydroxyl radical	OH
Singlet oxygen	^1^O_2_
Peroxyl radical	LOO
Alkoxyl radical	LO
Lipid hydroperoxide	LOOH
Peroxynitrite	ONOO^−^
Hypochlorous acid	HOCl
Ozone	O_3_

Reactive nitrogen species (RNS) and reactive chlorine species (RCS) also play roles in oxidative stress [54].

Due to the post-translational modification (oxidation and phosphorylation) of proteins and aberrant signaling, which are mediated by ROS, oxidative stress leads to cell and tissue damage [55].

ROS could activate mitogen-activated protein kinase (MAPK) family which include 9ERK1/2, p38 MAPK and JNK. They control proliferation and function of both vascular and cardiac cells [55,60]. Other molecular actions induced by oxidative stress, dysregulate cell signaling and cause inflammation, proliferation, apoptosis, migration and fibrosis. These processes lead to unpropitious changes in vascular function, cardiovascular remodeling, dysfunction of kidneys, overactivity of the sympathetic nervous system and activation of immune system by influence on tyrosine kinases (c-Src, PI3K/Akt, FAK) and receptor tyrosine kinases (VEGFR, EGFR, PDGFR) [56,61]. However, researchers are not consentaneous if inflammation is a reason or a consequence of AH.

As mentioned above, activated immune cells generate ROS through Nox-dependent mechanisms, which lead to cytokine production. Infiltration of immune cells into the vascular wall, kidney and heart is a reason of tissue damage in hypertension. ROS have an impact on the differentiation and function and lead to activation of the CD8+ cytotoxic T cells. They connect to a target cell, implement a perforin into the target cell’s membrane and kill the target cell with granzymes. Granzyme A could damage the target cell’s mitochondria by generating ROS within the cell’s cytoplasm. Moreover, both, CD8+ and CD4+ T lymphocytes cause the secretion of cytokines which increase levels of oxidative stress [62]. It should be noted that also aldosterone regulates ROS generation by non-T cell-dependent mechanisms, which indicate a correlation between oxidative stress and RAAS in hypertensive target organ damage [62].

RAAS, especially Ang II, directly or by escalating the amount of ROS, could increase renal cell proliferation. Importantly, the number of mesangial cells and fibroblasts correlates with stage of renal dysfunction [63].

**Figure 4:**
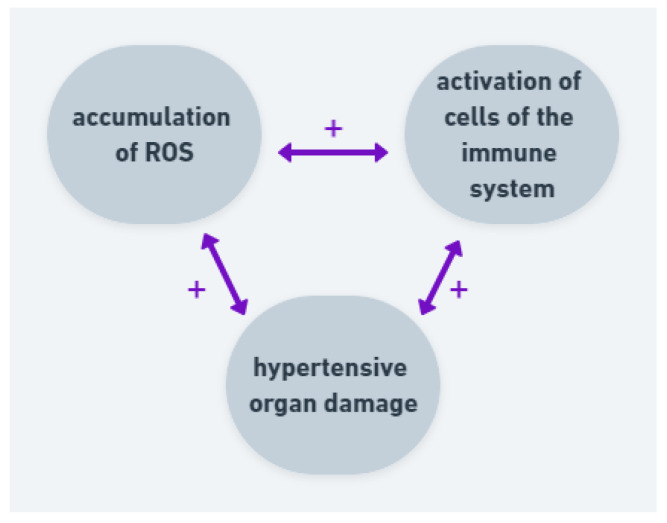
Correlation and positive feedback between ROS, immune cells and hypertensive organ damage. Accumulation of ROS leads to activation of cells of the immune system and both factors lead to hypertensive organ damage. The organ damage intensifies ROS production and activates immune system.

AH, cerebrovascular disease and dementia are strictly associated. Studies suggest that an excessive ROS generation has an impact on development of the neuronal death in brain disorders, probably through modulation of transmembrane potential and inhibition of ATP synthesis. Moreover, there are not many antioxidants in central nervous system [64]. All those factors are important in pathogenesis of AH and hypertensive organ damage. Researchers suggest that oxidative stress may be a more important factor among males than females [62,65]. The studies have characterized a relationship between oxidative stress, RAAS and activation of circulating immune cells (Figure 4). In the case of hypertension, the correlation between inflammation, ROS and blood pressure elevation is important in development of hypertensive organ damage.

## 6. Role of Endoplasmic Reticulum Stress in Hypertension and Its Target Organ Damage

The endoplasmic reticulum (ER) is the largest organelle in the cell. It is composed of various multiple domains, which are associated with calcium storage, protein synthesis and transport, as well as lipid and steroids metabolism and transport [66]. It is the first compartment of the secretory pathway in the cell. There are distinguished two types of ER: the rough ER (RER) and smooth ER (SER), depending on the presence or absence of ribosomes on the cytosolic face of the membrane.

The endoplasmic reticulum stress (ERS) is the state when the homeostasis of ER is disturbed. In order to maintain the balance, the cell turns on an adaptive signaling pathway called the unfolded protein response (UPR). The UPR’s main task is to help cells deal with the ERS by increasing protein synthesis, removing the incorrect proteins, together with rising the capacity of the ER [67].

Recently, there has been a great deal of evidence produced for the pathophysiological role of ERS major organs, such as the heart or kidney, in the development of hypertension.

This study investigated the role of ERS within the aorta. The induction of ERS leads to changes in the vascular smooth muscle cells (VSMC), as well as to its apoptosis and fibrosis. Moreover, there is developing data that the ERS response is associated with the rising BP in rats. As a consequence of ERS, the aorta stiffening and endothelial dysfunction occur; therefore, there is a greater risk of heart failure development [68]. Increasing evidence indicates that ERS and various compounds of UPR participate during cardiac remodeling and fibrosis [69]. Furthermore, these findings suggest that cardiac ERS is induced via an Ang II type 1 receptor-dependent pathway. Accelerated activation of ERS leads to cardiac myocyte apoptosis and, as a result, to the progression of heart failure [70].

Furthermore, there are developing data that the ERS induce pathogenic UPR pathways and that these cause the progression of various kidney diseases, especially acute kidney injury and chronic kidney disease. Moreover, recent research results show that the alteration of protein homeostasis, due to prolonged ERS, results in damage to the renal cells [71].

**Figure 5:**
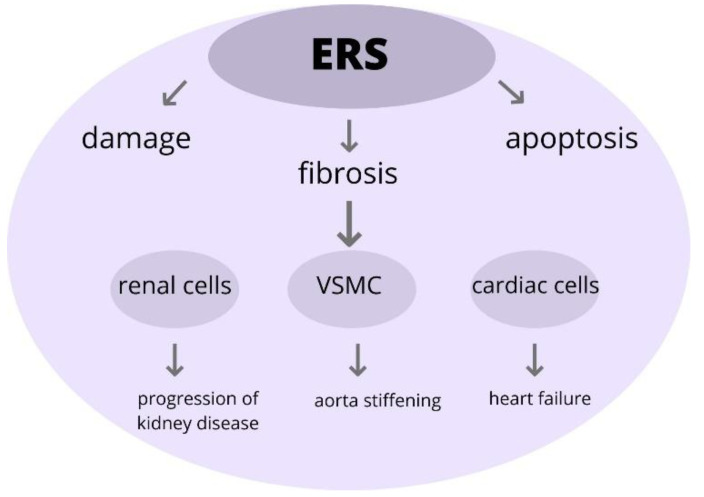
The role of ERS in pathophysiology in TOD. Excessive ERS can trigger pathological changes within the cells, leading to its injury and impairment and eventually participating in the onset of development and progression of organ damage. Abbreviations: ERS-endoplasmic reticulum stress, VSMC-vascular smooth muscle cells, TOD-target organ damage.

In conclusion, the development of TOD is associated with the upregulation of ERS. The ERS has a crucial role in the pathogenesis of various diseases and that might be a potential therapeutic agent for treatment (Figure 5).

## 7. Conclusions

Nowadays, high blood pressure is well recognized to play an important relative role in the pathogenesis of TOD, such as ischemic heart diseases, atherosclerosis and stroke, together with renal diseases.

AH remains one of the leading causes of CV disease and is correlated with high morbidity and mortality in all countries. AH is characteristically linked to vascular dysfunction, cardiovascular remodeling, renal dysfunction and stimulation of the sympathetic nervous system. TOD manifests as vascular injuries in body organ systems associated with chronic hypertension.

In our publication we want to discuss the molecular interactions in the pathophysiology of hypertension and show how they affect TOD.

Knowledge of how matrix metalloproteinases, the immune system, the renin-angiotensin-aldosterone system and oxidative stress are involved and their role in the pathophysiology of hypertension is not yet fully understood. Nevertheless, taking into consideration the currently available data, in this ork, we discuss these main molecular interactions.

One of the purposes of anti-hypertensive treatment is the prevention of end-organ damage. We believe that acknowledgment of the molecular abnormalities in hypertension and its impact on organ damage might become important in order to introduce new therapeutic tools for treating hypertensive patients.

## Figures and Tables

**Table 1:** Categories of BP [4].

CATEGORY	BP [mmHg]
Normal	systolic: <130 diastolic: <85
Elevated	systolic: <140 diastolic: <90
Stage 1 hypertension	systolic: 140–159 diastolic: 90–99
Stage 2 hypertension	systolic: 160–179 diastolic: 100–109
Stage 3 hypertension	systolic: ≥180 diastolic: ≥110

**Table 2:** The causes and prevalence of secondary hypertension [4,7].

Cause of Secondary AH	Prevalence
**Sleep apnea**	>5–15%
**Hyperaldosteronism**	1.4–10%
**Renal parenchymal diseases**	1.6–8%
**Renal artery stenosis**	1–8%
**Thyroid diseases**	1–2%
**Aortic isthmus stenosis**	<1%
**Cushing syndrome**	0.5%
**Pheochromocytoma**	0.2–0.5%

**Table 3:** Hypertension-related target organ damage [13].

Organ	Pre-Clinical/Clinical Damage
Heart	ischemic heart disease atrial fibrillation left ventricular hypertrophy heart failure sudden cardiac death
Heart valves	mitral/aortic valve calcification aortic valve sclerosis
Central arteries	aortic dissection decreased aortic compliance
Peripheral arteries	increased peripheral arterial stiffness peripheral atherosclerosis
Kidneys	proteinuria nephropathy decline in renal function chronic kidney disease end-stage renal disease
Eyes	retinopathy ischemic optic neuropathy hypertensive optic neuropathy choroidal neovascularization retinal vascular occlusion
Brain	hemorrhagic/ischemic stroke small vessel cerebral ischemic disease vascular dementia cognitive impairment

## Data Availability

The data used in this article is sourced from materials mentioned in the References section.
